# Deceptive nest defence in ground-nesting birds and the risk of intermediate strategies

**DOI:** 10.1371/journal.pone.0205236

**Published:** 2018-10-08

**Authors:** Paul A. Smith, Darryl B. Edwards

**Affiliations:** 1 Environment and Climate Change Canada, Wildlife Research Division, National Wildlife Research Centre, Ottawa, Ontario, Canada; 2 Cambrian College, Sudbury, Ontario, Canada; University of Reunion Island, RÉUNION

## Abstract

Nest predation is an important determinant of reproductive success and ground-nesting birds exhibit a variety of nest defence strategies to mitigate the risk. Many small-bodied, ground nesting birds rely on deceptive behaviours such as injury-feigning to reduce nest predation: we call this behaviour active deception. However, active deception may entail risks to adults, and passive deceptive behaviour, where individuals effectively sneak away from the nest by flushing at long distances, may be an alternative means of avoiding nest predation. We provide a simple model to demonstrate that these tails of the flush distance distribution could minimize predation risk; an intermediate strategy of moderate flush distances means that birds flush more often than with short-distance flushes, and once flushed, the nest is more easily located than for long-distance flushes. We tested this model using two species of ground nesting shorebirds, the White-rumped Sandpiper (*Calidris fuscicollis*) and the Red Phalarope (*Phalaropus fulicarius*). We demonstrate that short-distance flushes are associated with active deception and intermediate-distance flushes are associated with an increased risk of nest predation. However, we found no evidence that this potential selective pressure against intermediate strategies has produced a bimodal distribution of nest defence traits. The heritability of defence behaviours, or the ability of individuals to learn, is unknown and other factors such as energetic constraints or risks to adults might also influence flush distances and defence behaviours.

## Introduction

The role of predators in shaping the breeding ecology of birds cannot be overstated. Predators exert an overwhelming influence on reproductive success and are nearly always the largest source of nest failure for ground nesting birds [1–2]. Predation pressure has been shown to influence choice of nest site [3], optimal clutch size [4] and various aspects of breeding behaviour [5–6]. Indeed, predation is such a large determinant of reproductive success that tactics to mitigate nest losses to predators may be more important than other traits to increase fecundity [7]. An important behavioural adaptation to reduce the probability of nest predation is nest defence.

Many birds exhibit aggressive behaviours intended to drive a potential predator from the location of the nest or chicks. Aggressive nest defence is most common when the defending individuals pose a threat or nuisance to predators, typically because of a large body size [8], shared defence between two parents [9] or cooperation among nearby individuals [10]. The evidence for benefits of strong aggressive nest defence is clear. Increased defensive effort from the parents correlates with higher reproductive success ([11–15], but see [16]) and aggressive defence is often intensified when the value of a clutch to parents increases [7, 17] or when predators get nearer to the nest [18].

For many small-bodied birds with single-parent incubation, aggression is not a practical means of nest defence because the parents pose no risk to the predator (e.g., [8]). These species primarily utilize deceptive strategies, and we propose that there are two forms of deceptive nest defence: active deception and passive deception. When a predator approaches a nest and is detected, an incubating bird must decide whether to remain on the nest or to flush and leave the nest area. Many birds flush from the nest and perform a conspicuous distraction ploy, such as feigning a broken-wing or mimicking a rodent [19]. These distraction displays (“active deception”) are intended to focus a predator’s attention on the adult, thus luring the predator away from the nest site. Consequently, these active forms of deception are risky to adults, and in some cases fatal [20]. Moreover, predators may learn to associate the deceptive behaviours with the presence of a nest [21], so that in some situations it may be disadvantageous for a bird to attempt to distract the predator. Other birds will flush from the nest while the predator is still distant and attempt to leave the area unnoticed (“passive deception”), minimizing the risk to themselves. The deceptive behaviours available to ground nesting birds, ranging from passive to active, thus vary in terms of risks and potential rewards. While aggressive nest defence has been well studied, the balance between risks and rewards for these deceptive nest defence strategies has received little attention.

Shorebirds are common ground nesting birds of open habitats, and vary widely in the defence of their nests. Aggressive nest defence is common among larger bodied, monogamous shorebird species where both parents share in the duties of incubation (“biparental”), while deceptive strategies are more common in smaller bodied, polygamous species with single-parent incubation (“uniparental”; [9]). For species employing deceptive strategies, some individuals do not flush until the threat is within 1 m while others flush at 30 m or more ([22], this study). Some individuals attempt to depart unnoticed (passive deception) while others exhibit active deception behaviours including injury feigning (i.e., broken-wing display) or the “rodent run” display where the individual runs low to the ground while emitting a variety of high-pitched vocalisations. Here, we develop and test a model suggesting that both short-distance flushing with active deception and long-distance flushing with passive deception can help shorebirds evade predators, but that intermediate flush distances are disadvantageous. We also examine the distribution of observed behaviours for 2 uniparental shorebird species to test whether selection or learning has operated to reduce the frequency of intermediate behaviours in the population.

### A model of alternative strategies for deceptive nest defence

Movement around the nest can draw attention to its location and increase the risk of predation [5, 23–24]. A bird’s departure from the nest is a strong visual cue that reveals the nest location, and predators presumably use this cue (in addition to other cues) to locate and depredate nests. We assume that this cue can contribute significantly to the likelihood of nest predation; indeed, walking and watching for flushing birds is how many ornithologists locate birds’ nests. The model presented below considers the risk arising as a function of this cue. Other factors such as variation in nest habitat (e.g., [3]), olfactory cues (e.g., [25]), and adult or egg camouflage (e.g., [26]) could also influence predation risk, but are not considered here.

A bird that flushes from a long distance has an increased probability of being flushed by a predator simply because of the increased probability of a predator entering a circle with a large radius. This long-distance flush will not increase the risk of nest predation if the flushing bird is not seen by the predator, or if the predator cannot determine the exact location from which the bird flushed. If a long-distance flush does not reveal the nest location, it may be disadvantageous for a bird to then return to the nest location and exhibit active deception. In contrast, for birds that flush from a short distance, the predator is less likely to come within the threshold distance (i.e., a smaller circle) and flush the incubating bird. But at very short distances, it is much more likely that the predator will see the flushing bird and determine the exact location of the nest. Consequently, birds flushing at short distances might benefit more from active deception, to draw the predator’s attention away from the nest.

There are therefore potential advantages at both tails of the distribution from long-distance flushing with passive deception to short-distance flushing with active deception. However, behaviours between these tails may be disadvantageous. Intermediate flush distances may provide an optimal target for predators since they provide a compromise between the probability of flushing an incubating bird and the distance between the predator and the nest when the bird is flushed (i.e., influencing subsequent detection probability by the predator). If intermediate behaviours indeed increase the risk of nest predation, and these behaviours are heritable or can be learned, avoidance of intermediate behaviours might produce a bimodal distribution of behaviours.

**Fig 1:**
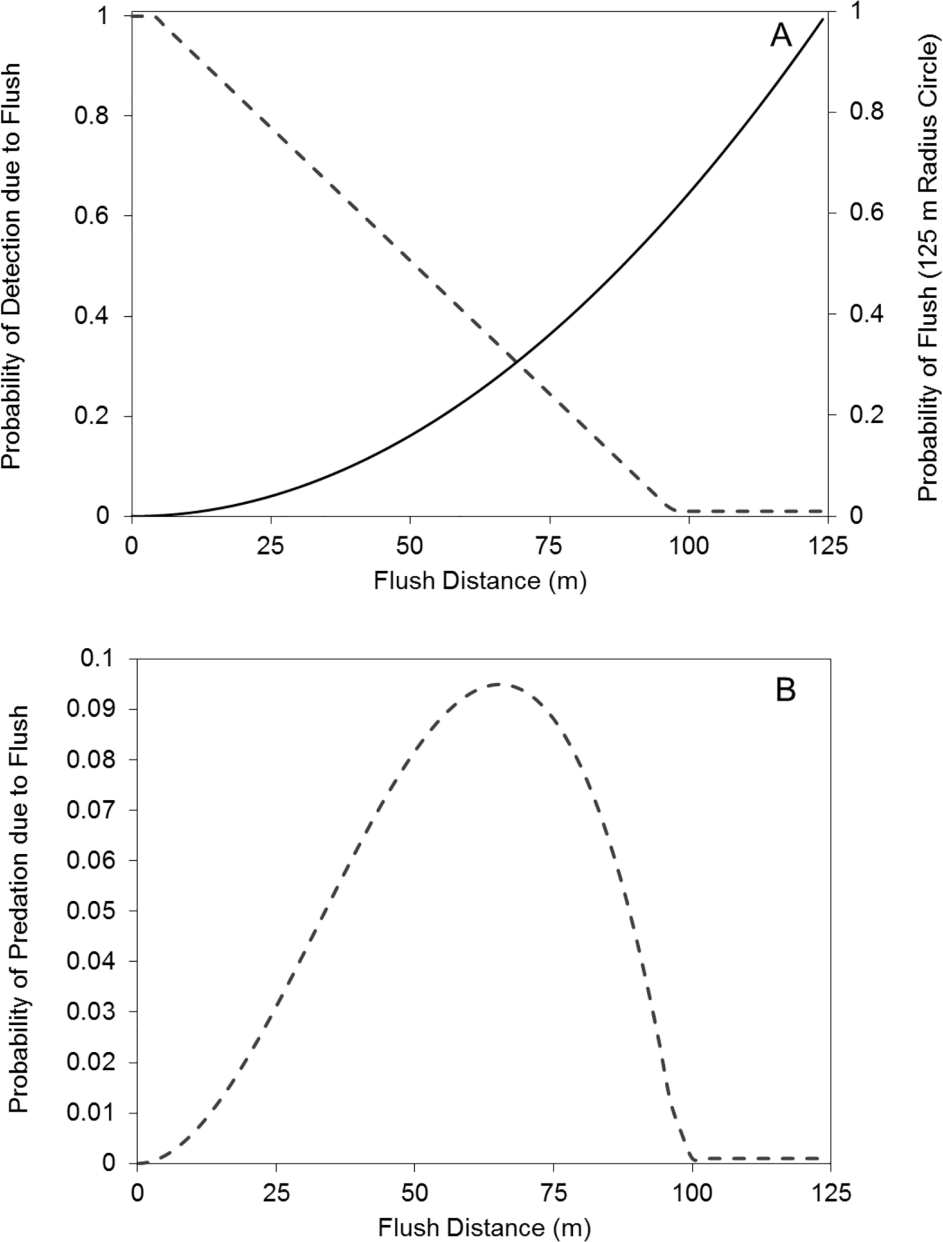
A model of the risk attributable to flushing from the nest. The likelihood of a predator observing a bird flush from the nest, and using this cue to find the nest, decreases from 100% at very small flush distances to 0% at very large flush distances. If we assume that predators will find all nests when birds flush at a distance of ≤ 5 m and that flushing at ≥ 100 m does not influence the risk of predation, the probability of nest detection is as shown in panel A. Birds flush when predators come within a threshold distance, thus the likelihood of flushing increases as the square of the threshold flush distance. Panel A shows this “probability of flush” given that a predator occurs somewhere within a 125 m radius of the nest (i.e., a bird that flushes from 125 m has a 100% probability of flushing if a predator is anywhere within 125 m of the nest). The risk function (panel B) is the product of these two probabilities, and shows that moderate flush distances are the most likely to contribute to predation risk.

We assume that a predator is certain to locate a nest if the bird is flushed at very close range (*F*_close_). At very long distances, the fact that a bird flushes from the nest should have no effect on the risk of predation (*F*_long_). Our observations as human nest searchers suggest that 5 m is a plausible value for *F*_close_ and 100 m is a plausible value for *F*_long_. We assume that the probability that a predator will locate a nest as a consequence of the bird’s flush declines from 100% at *F*_close_ to 0% at *F*_long_. If we assume that a bird will flush when a predator enters a circle with a radius equal to the bird’s threshold flush distance, the probability of a bird flushing increases with the square of the threshold distance. These parameterizations of our simple model are shown in Fig 1A. The product of the detection and flush probability curves reveals high risk for birds flushing at intermediate distances (Fig 1B). Given our assumed values for *F*_close_ and *F*_long_, birds suffer the greatest risk of nest detection as a consequence of flushing when they flush at distances of 30–80 m. Adjustments to our assumed values affect only where the maximum risk lies, but does not alter the interpretation that intermediate flush distances maximize the likelihood of a predator locating the nest as a consequence of the flush.

## Materials and methods

All procedures were approved by the Animal Care Committee of Environment and Climate Change Canada, and through permits from the Federal and Territorial Governments (e.g., 2006PNR008, WL00069). Because this was an observational study, there were no significant ethical concerns relating to animal welfare.

We evaluated this model by collecting field data from 2 species of breeding shorebirds in 2005–2006 and 2008–2009, at the East Bay Migratory Bird Sanctuary (N63° 59’ W81° 41’), Nunavut, Canada. Within the 12 km^2^ field site, we studied the breeding behaviour of White-rumped Sandpipers (*Calidris fuscicollis*) and Red Phalaropes (*Phalaropus fulicarius*), hereafter referred to as sandpipers and phalaropes. Both species nest in dry upland hummocks, moist sedge meadows and pond edge habitats; the nest habitat preferences of these species are described in detail in Smith et al. [27]. Both sandpipers and phalaropes are polygamous with only one parent providing care to the young, including during incubation. For the polygynous sandpiper the female incubates whereas males incubate in polyandrous phalaropes. Neither species uses aggression to defend their nests; they rely on camouflage to conceal the nest site and for some individuals, active deception to lead predators away.

Due to the coastal location of the East Bay study site, and the strong influence of cool currents from the Foxe Basin, vegetation at the site is stunted and rarely grows taller than 15 cm. Arctic foxes (*Alopex lagopus*) are the major predators of shorebird nests along with Parasitic Jaegers (*Stercorarius parasiticus*). Long-tailed Jaegers (*Stercorarius longicaudus*) and Herring Gulls (*Larus argentatus*) are likely less important, but present at the site [17].

Nests were located through systematic searches of 1 km^2^ plots within the 12 km^2^ study area. Nests were located by either observing individuals return to the nest after feeding or by flushing incubating birds while walking (this nest searching method could increase the frequency of moderate distance flushes in our sample, see Discussion). Upon finding a nest, we placed a small tongue depressor several metres away and recorded coordinates with a handheld GPS unit. Nests were aged using the flotation method (accurate ± 4 days in most cases; [28]).

All nests were revisited approximately every seven days to determine nest fate. Float information was used to predict hatch dates and as this date approached, nests were visited every other day to avoid missing the hatch. Nests were considered successful if one or more eggs hatched. If nests were found unexpectedly empty near the hatch date, nest contents were sifted to search for egg shell fragments that would confirm a successful hatch [29]. In addition, the area surrounding the nest was searched for large egg shell fragments or signs of other predator activity (e.g., defecation).

To examine the behavioural response of shorebirds to a simulated predator, a human observer approached nests from a random bearing at normal walking speed (~ 4km/h). Flush distance was measured as the distance between the observer and the nest when the bird first left the nest, and was measured either by pacing or with a handheld GPS unit for longer distances. We then approached the nest and remained there for a period of two minutes during which time the birds’ behaviour was recorded as: 1) pure flush (passive deception)—the bird flushed off the nest and performed no other defence behaviour; 2) active deception involving either a rodent-run or broken-wing type distraction display; 3) active deception incorporating calls, interpreted as an escalation in the distraction efforts of the incubating bird; 4) attention flights—hovering by the observer and calling, typically used in conjunction with other distraction displays (i.e., categories 2, 3). These nest defence behaviours were recorded on the second visit to the nest (i.e., the first visit after finding) in order to standardize the number of human induced flushes of the incubating bird. We have previously shown that flush distance and defence behaviour do not change with nest age in uniparental species [17], as was the case in this dataset (linear regression of age vs. flush distance, R^2^ = 0.005, age vs. behaviour, R^2^ = 0.03).

To determine whether flush distances deviated from a unimodal distribution, we used the dip test [30–31] implemented in package ‘diptest’ [32] in R 3.3.1 [33]. This test looks for departures from unimodality based on 'dips’ in the cumulative frequency distribution.

We determined whether categorical nest defence behaviours were related to flush distances using ordinal regression and examined the relationship between flush distance and nest fate (successful vs. depredated) using binomial logistic regression. Flush distance was compared between species with a t-test. Analyses were carried out with R 3.3.1.

## Results

We found a total of 102 sandpiper and 74 phalarope nests during the four years of study. Apparent hatch success (successful nests/total nests) was 12% for sandpipers and 19% for phalaropes. Fate was unknown (fate could not be determined or the nest was still active when we departed) for an additional 7% of sandpiper and 9% of phalarope nests. More complete analyses of nest survival are available elsewhere (e.g., [17]). We were able to record flush distance during the second visit for 100 nests (62 sandpipers, 38 phalaropes; S1 Table). Uniparental incubators take regular incubation recesses [23,34] and for some nests we were unable to record flush behaviours because the bird was not incubating at the time of the visit. In other cases, the nest failed in the short time between finding it and returning to collect the flush data, or the flush was simply not seen (the latter could not be distinguished from off-duty birds, and is presumably more likely to have occurred for longer-distance flushes).

Sandpipers displayed the full range of categorical nest defence behaviours from a pure flush to attention flights in conjunction with calls and other forms of distraction display (Table 1). The modal defence category in sandpipers was 2 whereas in phalaropes it was 1; sandpipers most often carried out a rodent-run distraction display while phalaropes most often flushed from the nest and left the immediate area, often visiting a pond to feed and preen. Active deception in phalaropes, when it occurred, was limited to calls and a one- or two-winged paddling motion which was less energetic and less elaborate than the rodent-run or broken-wing display of sandpipers.

**Table 1:** The proportion of individuals displaying each category of deceptive behaviours.

	Proportion of behaviours in each category			
Species	1) Pure flush	2) Distraction display	3) Distraction display with calling	4) Attention flights
**Red phalarope**	0.84	0.08	0.08	0.00
**White-rumped sandpiper**	0.26	0.28	0.34	0.11

**Fig 2:**
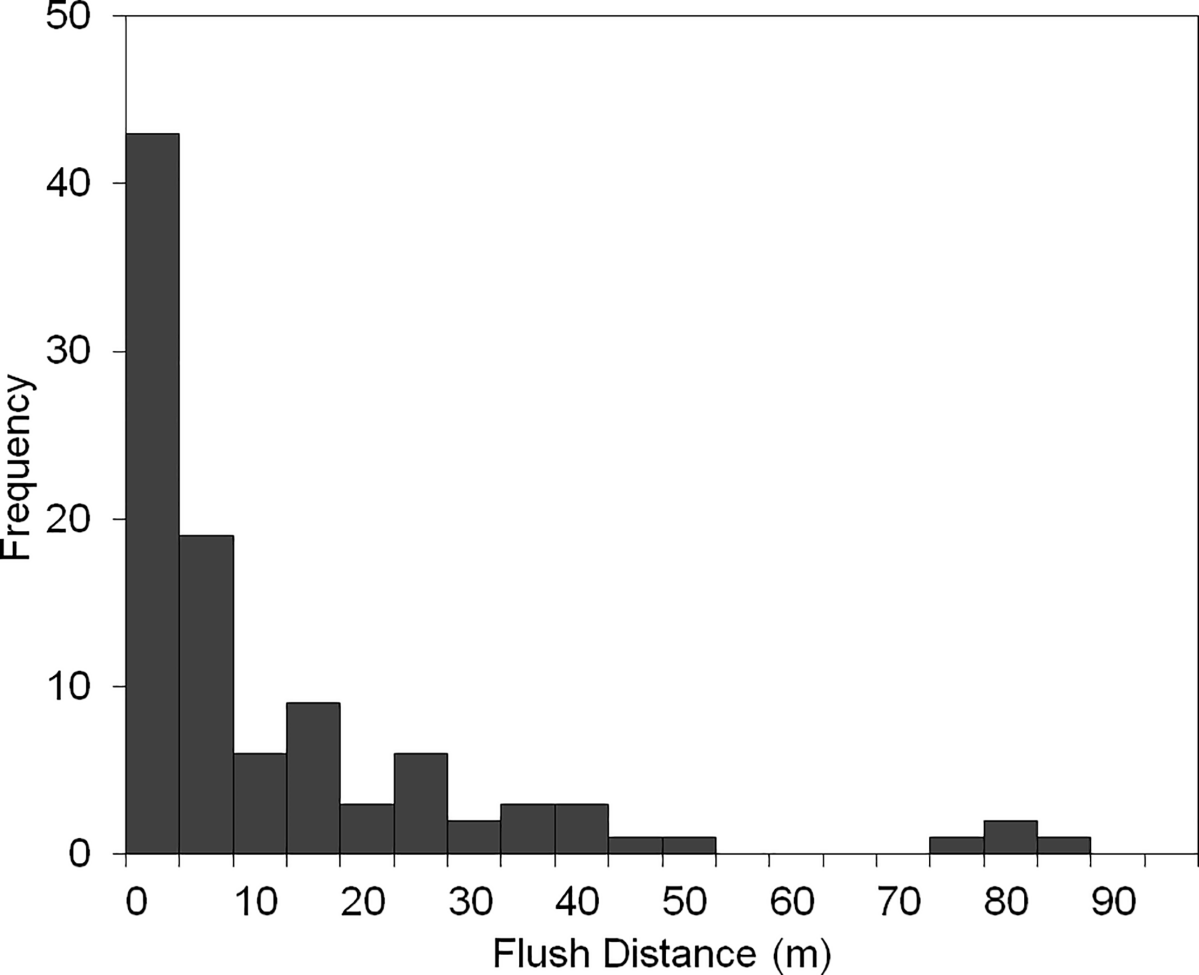
Observed flush distances. The observed distribution of flush distances from our sample of incubating Red phalaropes and White-rumped sandpipers.

Flush distance was highly variable, ranging from 0–86 m. It did not differ between species (*t*_*98*_ = 0.38, *P* = 0.47), and the mean ± SD was 15.3 ± 18.8 m for sandpipers and 13.8 ± 19.0 m for phalaropes. The dip test revealed no significant departure from unimodality (D = 0.03, *P* = 0.78); 41% of individuals flushed from <5m, 36% from 5-20m and 23% from >20m (Fig 2).

**Fig 3:**
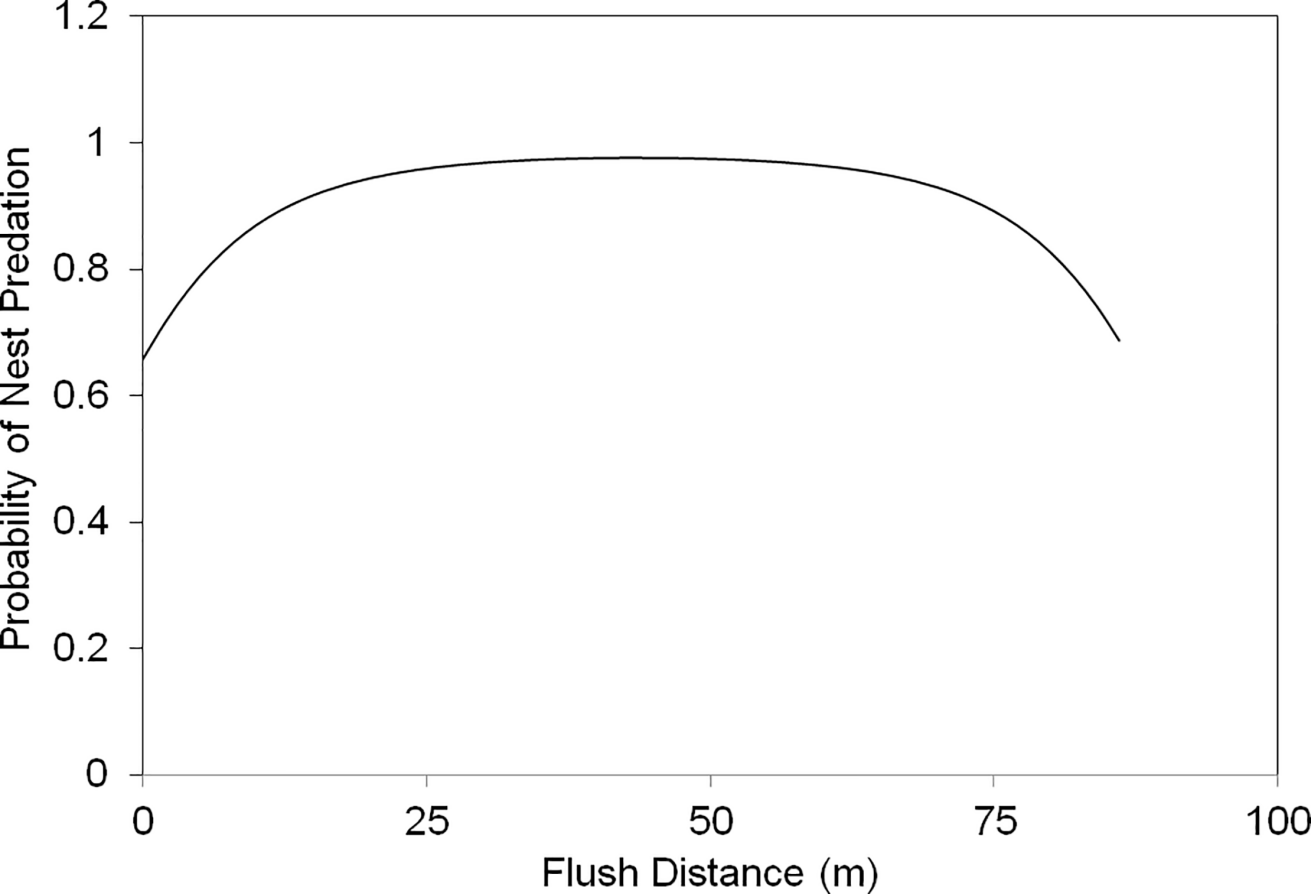
Flush distance versus nest survival. Model predictions from a binomial logistic regression of nest success versus flush distance and flush distance^2^.

Birds with short flush distances exhibited stronger nest defence. After accounting for interspecific variation, we found that flush distance was a significant predictor of the behavioral defence category (Species: *Χ^2^* = 28.3, *P* < 0.0001, Flush Distance: *Χ^2^* = 5.3, *P* = 0.021), with short flush distances associated with the more intensive categories of active deception. The probability of nest predation was highest for intermediate flush distances. Flush distance and flush distance^2^ were significant predictors of nest fate (Flush: β ± SE = -0.141 ± 0.065, *P* = 0.030, Flush^2^: 0.002 ± 0.0008, *P* = 0.036). Nest survival varies across species and years in larger datasets at this site, but these variables were not significantly related to nest fate in this sample (Species: *P* = 0.106, Year: *P* = 0.332). Inclusion of species and year effects did not alter the significance or the interpretation of the Flush and Flush^2^ effects. Predicted values from the logistic regression suggest that the risk of predation was lowest for very short and very long-distance flushes, and elevated for intermediate distances (Fig 3).

## Discussion

We hypothesized that predators’ perception of flushes is an important component of the nest predation process, and predicted that intermediate flush distances should increase the risk of predation because they occur more frequently than short-distance flushes and are more conspicuous than long-distance flushes. We also predicted that active forms of deception should be more commonly associated with short flush distances, because the risks of these behaviours are justified only when predators are in close proximity to the nest. Finally, we proposed that this could create a bimodal distribution of flush distances in the population if flush behaviour is heritable or can be learned. As predicted, we found evidence that intermediate flush distances were associated with a higher risk of nest predation, and active forms of deception were associated with short flush distances. However, very long-distance flushes were rare in our dataset and we found no strong evidence for bimodality in the distribution of flush distances.

Our data suggest that the risk of nest predation was greatest for phalaropes and sandpipers that flushed at distances between 20–70 m. The distribution of flush distances in our sample was skewed towards shorter distances, with the median flush distance less than 10 m. Our regression results suggest that the risk of predation was again lower for flush distances greater than 80 m, but we rarely observed flushes at such long distances (3 observed flushes were >80 m). Some shorebird species, such as *Pluvialis* plovers, flush at distances as far as several hundred metres [19]. In these species, nest site selection often favours locations with greater visibility of the surroundings allowing these longer-distance flushes [27,35]. In contrast, phalaropes and sandpipers actively select concealed nest sites [27] and in a previous study at this site, flush distance was found to be shortest for the most concealed nests in a small sample of phalaropes [22]. While we acknowledge that the longest flushes are more likely to be missed by us, we regularly observe flush distances >100 m in (biparental) plover species at East Bay. We feel that it’s unlikely that sandpipers and phalaropes regularly flush at distances >80 m, but that these flushes were missed by us. Instead, we suggest that a strategy of very long-distance flushing (i.e., >80 m) to avoid detection by predators might be unavailable to most sandpipers and phalaropes because the concealment of their nest sites does not allow them to detect predators from these distances.

A strategy of very long-distance flushing is successful for some shorebird species, however. Byrkjedal [36] reported that a species of plover flushing from long distances (*Pluvialis apricaria*) had higher hatch success than a sympatric plover species (*Charadrius morinellus*) that is uniparental and uses injury feigning displays to deceive predators at short distances. Similarly, Koivula and Rönkä [37] suggest that the typical strategy of stints (*Calidris temminckii*), which is a long flush and no display, may be particularly effective against predators that use olfaction to detect nests. Our model is restricted to the visual component of the nest defence process, which could differ in importance among predators. Avian predators such as jaegers use visual cues primarily, while foxes use olfaction in part [38], but the relative importance of olfactory versus visual cues is unknown. Regardless of this relative importance, the observation that birds vary in flush- and defence-behaviour when confronted with foxes (e.g., this study) suggests that foxes do respond to visual cues.

Alternatively, long flushes (and therefore more frequent flushes) could be disadvantageous if the time spent off the nest prolongs incubation or results in reduced hatchability. Also, studies have suggested that shorebird nests left uncovered for greater proportions of the day may suffer a higher risk of predation [23], perhaps because eggs are more visible to avian predators than incubating adults. Our model does not account for these potential influences on nest survival; it explores only the component of risk arising from the act of flushing.

We predicted that individuals that flushed at short distances would display the strongest nest defense behaviours. Selection for strong defence is typical for bird species that aggressively defend their nests [11–14]. We suggest that the potential benefits of active deception are greatest when predators approach the nest and present a more immediate threat to the clutch. Consistent with our predictions, we found that individuals flushing at long distances left the vicinity of the nest without performing distraction displays, while individuals flushing from closer distances were more likely to engage in injury feigning or other forms of active deception. In our study, this short flushing/active deception strategy was associated with reduced risk of nest predation, suggesting that the distraction behaviors were effective. However, this strategy may be less effective if predators learn to ignore such displays.

Sonerud [21] observed occasions when foxes used the presence of a displaying hen grouse (*Tetrao tetrix*) as evidence of chicks nearby. The fox identified a search location because of the nest defence behaviour of the female, and ignoring her displays, intensely searched the surrounding area before locating chicks. Sonerud [21] makes the argument that it is most advantageous to use nest defence displays on naïve foxes while it may be disadvantageous for foxes that have learned to associate the behaviour with a reward. He then supports his model with empirical evidence that suggests there are fluctuating benefits to distraction displays based on the demographic structure of fox populations. Arctic foxes are the dominant nest predator of Arctic Charadriiformes [35,39–40], and their local population dynamics are influenced by fluctuating abundance of small mammals, so similar variability might be expected in the benefits of deceptive nest defence behaviours in this system.

Our failure to detect bimodality in the distribution of flush distances could reflect fluctuating benefits such as this, weak heritability or learning of appropriate flush distances, or most likely, bias in our sample. We searched for nests by walking the tundra and looking for flushing birds; we are most likely to find nests with moderate flush distances for the very reasons explored in our model. We are also more likely to observe short flushes and moderate distance flushes than very long-distance flushes. These biases serve to weaken our ability to test for bimodality in the data, but do not adversely affect our tests of the relationship between flush distance and predation risk, nor flush distance and deceptive behaviour.

The model we present here reflects the risk of predation arising from the act of flushing itself, not the overall risk of predation. As noted above, flushing behaviour can indirectly influence nest predation or the energetic condition of adults in more complex ways. Still, understanding the repertoire of behaviours available to shorebird species, how these are structured and how they influence reproductive success, is important to make sense of shorebirds’ varied mating systems. Our results suggest that sandpipers and phalaropes benefit from short-distance flushing and distraction displays, but may be constrained from employing strategies of long-distance flushing because of their concealed nest sites. Direct tests of nest concealment in relation to flushing distance, and tests across a broader suite of ground-nesting species, could help to resolve these relationships further.

## Supporting information

S1 Table Nesting and defence behaviour data. Dates of nest initiation, dates and values for flush distance and defence behaviour measurements, and fate for nests included in analyses.
(XLSX)
